# Mutagenic, Carcinogenic, and Teratogenic Effect of Heavy Metals

**DOI:** 10.1155/2022/8011953

**Published:** 2022-10-05

**Authors:** Sukeerthi Dasharathy, Selvam Arjunan, Anusha Maliyur Basavaraju, Vidya Murugasen, Saravanan Ramachandran, Rohini Keshav, Rajadurai Murugan

**Affiliations:** ^1^Department of Biotechnology, Faculty of Life and Allied Health Sciences, Ramaiah University of Applied Sciences, Bangalore 560054, Karnataka, India; ^2^Department of Food Technology, Faculty of Life and Allied Health Sciences, Ramaiah University of Applied Sciences, Bangalore 560054, Karnataka, India; ^3^Proiuvo Private Limited, Bangalore, Karnataka, India; ^4^Department of Marine Pharmacology, Chettinad University, Kelambakkam, Chennai, Tamil Nadu, India

## Abstract

Heavy metal (HM)-induced toxicity and its associated complications have become a major issue in the medical world. HMs are not biodegradable, enter into the food chain, and gets accumulated in the living systems. Increased concentrations and accumulation of HMs can cause severely damaging effects and severe complications in living organisms and can even lead to the death of the organism. In Ayurvedic medicine, ingredients of natural origin, including whole plants or certain portions of the plant, animal sources, and minerals, are used for therapeutic purposes as medicine, both alone and in combination. HM such as cadmium, copper, zinc, lead, chromium, nickel, and arsenic cause hazardous effects on animals, human health, and the environment. This review focuses on mutagenic, carcinogenic, and teratogenic effects of HM , mechanism, organ toxicity, available remedies in the market, and their side effects. Also, emphasis is given to alternative systems of medicine to treat HM toxicity.

## 1. Introduction

The naturally occurring metals with relatively high density are called as heavy metals (HMs). These metals with metalloids like arsenic are toxic and can cause health effects. Due to the increase in the application of these metals in different fields, including agriculture and industries, exposure to these harmful chemicals has increased drastically; it has become a global health concern [1].

**Figure 1:**
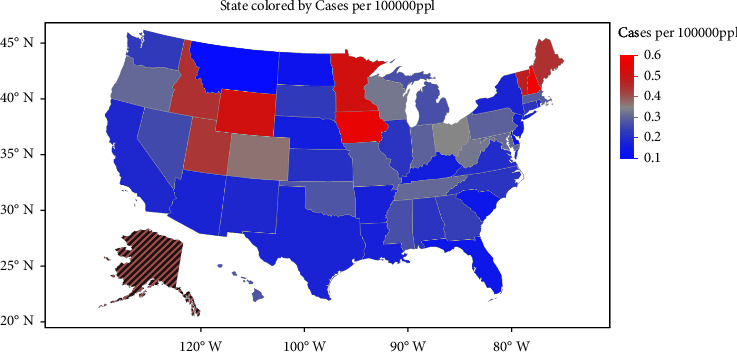
Severity of heavy metal (HM) toxicity cases (lead, arsenic, and mercury) in the USA. Adapted and redrawn from data available from the National Capital Poison Centre.

**Figure 2:**
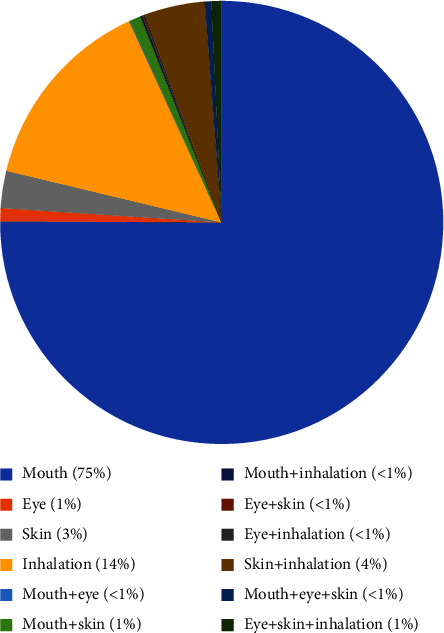
Heavy metal (HM) toxicity concerning the route of entry. Adapted and redrawn from data available from the National Capital Poison Centre.

Though these HMs are present in trace amounts in the environment, the exposure to these metals increases as the mining and smelting activities grow. Further, the use of these metals in many industries, including agriculture, and pharmaceutical also increases, which also leads to human exposure.  Figure 1 shows the severity of HM toxicity cases in the US. Environmental contamination of these metals can also occur due to corrosion, leaching of HM, soil erosion, and atmospheric deposition [2].  Figure 2 shows the statistical reports of HM toxicity concerning the route of entry.

These metals including arsenic, lead, cadmium, and mercury are nonessential elements and interact with different cell components like cell membrane, proteins, mitochondria, and DNA, causing reactive oxygen species (ROS)-related damages, transcriptional changes, DNA damage to cells leading to many diseases, and disorders like multiorgan damage and cancer [3].

The mechanism of toxicity differs with different HMs. Hence, understanding the exposure and toxicity of different metals is important for curing diseases and reducing the pollution caused by HMs. It's not possible to discuss and review all the HMs and their hazardous effects. Hence, we decided to concentrate on lead, mercury, and arsenic. This review focuses on mutagenic, carcinogenic, and teratogenic effects and its mechanism action of HM toxicity and complications associated with specific organs, and it also deals with the available remedies for HM toxicity and alternative system of medicine to treat HM toxicity.

## 2. Methodology

Required data were searched/collected from the online data bases including Wiley, Google, PubMed, Google Scholar, ScienceDirect, and Scopus. Keywords used in this search are HM-induced toxicity, alternative system of medicine, lead, mercury, cadmium, and mutagenicity, carcinogenicity, and teratogenicity. Latest published data were selected [3].

## 3. Lead

Lead has a wide range of applications in industries, agriculture, and domestic uses because of its physicochemical properties such as softness, high malleability, ductility, low-melting point, poor conductibility, and corrosion resistance. It is one of the oldest known occupational and environmental toxicants which is not biodegradable. The International Agency for Research on Cancer (IARC) has distinguished inorganic lead compounds under group 2*A* (probable human carcinogens), whereas lead metal under group 2*B* (possible human carcinogen) [4]. On exposure to lead, it accumulates in bones and teeth over a decade. Although the usage of lead has been discontinued in many parts of the world, it is still being used for smelting and manufacturing batteries. It is one of the highly toxic metals which affects major organs in the body [5]. The present review focuses on the sources, occupational exposure, mechanism of toxicity, available remedies, and alternate system of medicine.

### 3.1. Occupational Exposure and Sources

Elemental pollution is due to many man-made activities like industrialization, mining, and manufacturing. Lead has been used for the production of lead-acid batteries, ammunitions, oxides for paint, glass, pigments, chemicals, and devices to shield X-rays. The exposure to lead is being controlled by the leading industries (battery manufacturers), but there are some industries (demolition industry) where the exposure still occurs occasionally. Lead is found naturally in mineral deposits and they combine with the environment through industrial activity [6]. Lead poisoning can occur majorly from two routes, that is, inhalation and ingestion. Inhalation of lead can be through fumes or lead dust produced by industrial activities such as automobiles, smelting, storage batteries production, and lead-glazed ceramics. Cigarette smoking also releases lead. Ingestion through consumption of drinking water or food contaminated with lead enters into the food chain. The water pipes and solder made of lead may leach into drinking water. Children are more vulnerable to lead toxicity than adults. They are exposed through toys, deteriorated paint, dust, or bare contaminated soil. Automobiles emit lead components that are absorbed by the plants. Also, increased urbanization has led to an increase in the lead content in the sewage sludge and soil, thereby entering into the food chain [7]. Exposure to lead during pregnancy may be detrimental as the lead absorbed can be transferred to the fetus [1]. Dogs and cats are the most affected animals in urban areas [8]. The intake of polluted food sources, such as aquatic animals consuming lead-affected sediments in mining sites, can expose avian species to high levels of lead. Increased oxidative stress and reduced antioxidant enzymes in hepatic and renal tissue are two toxicities seen in these avian species [9].

### 3.2. Mechanism of Toxicity

**Figure 3:**
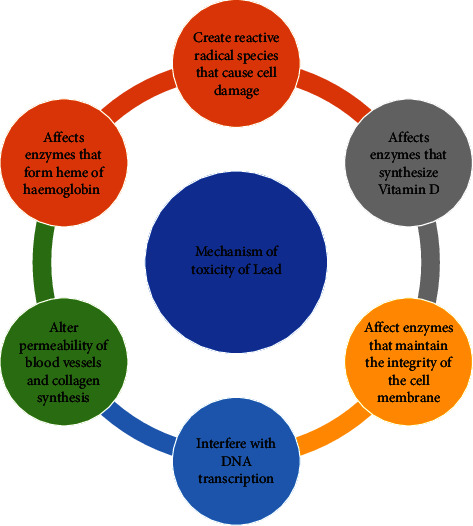
Mechanisms of toxicity of lead.

Various studies have been reported the different mechanisms by which lead exerts its toxicity, one of them being through oxidative stress. Exposure to lead causes oxidative stress, that is, an imbalance in the generation of ROS and antioxidant defense to nullify it [10]. The onset of oxidative stress is depicted by two pathways that occur simultaneously: (a) depletion of antioxidant reserves and its removal and (b) generation of ROS. Glutathione is a major intracellular antioxidant that can occur in both reduced (GSH, 90%) and oxidized (GSSG, glutathione disulfide, 10%) forms under normal conditions. Lead forms covalent attachment by electron sharing with sulfhydryl groups of antioxidant enzymes such as glutathione, glutathione reductase (GR), and glutathione peroxidase (GPx) which are the targets for inactivation [7].  Figure 3 shows the mechanism of toxicity of lead. Lead causes oxidative stress in the hepatocytes by raising free radical levels, causing lipid peroxidation, and lowering a number of antioxidants (MDA, SOD, etc.). NOX2 and CYP2E1 are two examples of genes whose transcription is disrupted by lead. Lead alters DNA, DNA methylation, mitochondrial stress, the electron transport chain, the IRE1-JNK pathway, RER damage in the ER, nuclear pyknosis, inflammation, and overexpression of NF-*κ*B, alterations in CYP7A1 gene expression, and HMGR, which alters cholesterol metabolism and causes hepatocytic necrosis [11].

The second possible mechanism of lead exerting its toxicity on the hemopoietic system is through *δ*-aminolevulinic acid (ALA). ALA is a molecule that is part of porphyrin synthesis leading to heme synthesis. In the mitochondria, it is formed by the condensation of glycine and succinyl-CoA by the enzyme ALA synthase. *δ*-Aminolevulinic acid dehydratase (ALAD), a cytosolic enzyme catalyzing the formation of porphobilinogen [12]. Lead can inhibit ALAD causing accumulation of ALA, generating free radicals such as superoxide (O_2_^−.^) and hydrogen peroxide (H_2_O_2_) radicals. The generated free radicals interact with oxyhemoglobin, generating hydroxyl (^•^OH) radicals. Increased lipid peroxidation causes oxidation of hemoglobin followed by hemolysis. Lipid peroxidation is a biomarker for oxidative stress. Lead can bind to phosphatidylcholine in the cellular membrane altering its properties [13].

### 3.3. Organ Toxicity and Associated Complications

The adverse effect of this compound is based on the level of exposure. Acute exposure although uncommon or through occupational exposure can affect the gastrointestinal tract, kidney, and brain, are more common, and meanwhile, chronic exposure has adverse effects on the nervous system, hematopoietic, renal, cardiovascular, and reproductive systems [14]. The nervous system plays a crucial role in the normal functioning of the body, and it is prone to be affected by lead toxicity due to its sensitivity. Lead affects both the central nervous system (CNS) in children and the peripheral nervous system (PNS) in adults predominantly. Acute encephalopathy is a condition associated with lead exposure to CNS, and some of the major symptoms are dullness, irritability, poor attention, muscular tremor, loss of memory, and hallucination [15]. At a lower level, fetuses and young children are vulnerable to lead toxicity as the lead can enter the blood brain barrier (BBB) resulting in hyperactivity and irritable behavior in them, while at a higher level it can cause permanent brain damage and death [16].

The hematopoietic system is affected by lead as low as 10 *μ*g/dl in the blood. Lead restrains the synthesis of hemoglobin, affects the heme biosynthetic pathway, and reduces the lifespan of erythrocytes as they become fragile leading to anemia. Hemolytic anemia (acute high-level lead exposure) and frank anemia (blood lead level elevated for a long duration) are caused by lead toxicity. Lead affects three key enzymes involved in the heme biosynthetic pathway, that is, aminolevulinic acid synthetase (ALAS), *δ*-aminolevulinic acid dehydratase (ALAD), and ferrochelatase [17].

The renal system is affected by exposure to lead as low as 10 *μ*g/dl and causes renal dysfunction at a concentration above 60 *μ*g/dl [18]. The contribution of lead to the cardiovascular system remains unclear, but a positive correlation has been reported between blood lead levels and hypertension [19]. Studies indicate that lead exposure decreases the bioavailability of nitric oxide, increases ROS production, and changes cytokine production which support atherogenesis and plaque development from elevated cholesterol levels [20]. An adverse effect of lead can affect the reproductive system in women and men. In women, lead exposure can make them susceptible to miscarriage, infertility, premature delivery, pre-eclampsia, and low birth weight of child born. Lead present in the blood can enter the fetus through the placenta as well as through breast milk increasing the toxicity. Exposure to low doses of lead in men reduces their sperm count, while exposure to high doses can cause a reduction in the sperm count and motile sperm number. Studies reported that lead can act as an endocrine disruptor molecule, i.e., it can delay puberty and alter menopause onset [21].

Lead toxicity is known to impair the immune (innate and adaptive) system. Occupational exposure to lead causes the generation of ROS in neutrophils and affects eicosanoid metabolism in the mature macrophages indicating its negative aspect on the innate immune system [22]. Lead affects the adaptive immune system through *T*-lymphocytes by interferring with the critical *T*-helper 1 (Th1)/*T*-helper 2 (Th2) lymphocyte balance required for resistance against infectious diseases. It reduces the CD^4+^*T* helper cells and increases the expression of 2,3-dioxygenase required for N-formyl-kynurenine formation. N-formyl-kynurenine is a metabolite of tryptophan metabolism causing apoptosis of *T*-lymphocytes and cell-cycle arrest explaining the depletion of CD^4+^*T* cells [23].

### 3.4. Treatment and Alternate System of Medicine

Chelation therapy is the treatment of choice to remove HM-induced toxicity. It involves chelate formation and excretion from the body upon administrating a chelating agent that binds lead [24]. The chelating agents are calcium disodium ethylenediamine tetraacetate (CaNa_2_EDTA) or dimercaprol and for oral chelation, it is D-penicillamine or *meso*-2,3-dimercaptosuccinic acid (DMSA). British Anti-Lewisite (BAL) has been used to treat severe lead poisoning where it evades precipitation of encephalopathy. Succimer (DMSA) is an oral chelation agent approved by the United States Food and Drug Administration (USFDA) effective in treating lead poisoning in children with BLL between 45 and 69 *μ*g/dl. The side effects associated with DMSA are an increase in hepatic transaminase, loss of appetite, nausea, and diarrhea. An individual with glucose 6 phosphate dehydrogenase (G6PD) deficiency should not be administered succimer as it can cause hemolytic anemia. Although chelation therapy has been the method of choice for the removal of lead from the body, it causes renal damage along with the loss of essential metals such as zinc, iron, and manganese [25].

Dietary strategies to overcome lead toxicity by excretion of lead involve supplementation of iron, zinc, calcium, and thiamine supplementation. Essential metals reduce lead burden by competing with them for intestinal absorption and binding to enzymes' active sites. Zinc supplementation protects the blood ALAD and alleviates oxidative stress as zinc is a cofactor for copper zinc-superoxide dismutase (Cu/Zn SOD), an antioxidant enzyme. Selenium also has a protective effect against lead toxicity as it contributes to the antioxidant defense by being a cofactor of GPx [26]. The deficiency of thiamine, pyridoxine, and ascorbic acid increases the sensitivity toward lead toxicity. The pyrimidine ring of thiamine interacts with lead, thereby mitigating its toxicity and increasing its excretion from the body. The nitrogen atom of pyridoxine ring chelates lead before its absorption, reducing its accumulation in the tissues and reducing inhibition of ALAD activity. Ascorbic acid and vitamin E have a positive impact on reducing BLL as it is a natural nonenzymatic antioxidant capable of attenuating oxidative damage [27]. Organosulphur compounds (diallyl sulfide and diallyl tetrasulfide) found in alliums such as garlic, ginger, and onion prevent intestinal absorption of lead and promote its excretion [28]. *Lactobacillus, Bifidobacterium, Saccharomyces,* and *Bacillus* are some of the commercial probiotic strains. *Lactobacillus rhamnosus, Lactobacillus plantarum*, and *Bifidobacterium longum* are some of the lactic acid bacteria (LAB) which bind HMs *in vitro*. The protective effect of *Lactobacillus plantarum* CCFM8661 against lead toxicity has been reported where they were found to reduce the BLL and prevent the oxidative stress caused by the metal [29].

## 4. Arsenic

### 4.1. Occupational Exposure/Sources

Arsenic has been employed for a variety of purposes throughout history, from medicines to poisons. Arsphenamine (marketed as Salvarsan) was used to cure syphilis until antibiotics were developed in the early 1900s; it was also used to treat trypanosomiasis and amoebic dysentery. Rheumatism, arthritis, asthma, malaria, trypanosome infection, TB, and diabetes were all treated using medicinal solutions such as potassium arsenite, arsenic iodide, and arsphenamine [29]. Arsenic contamination in humans can occur via arsenic-containing industrial effluent, agricultural pesticides, or natural mineral deposition in soil, air, food, and drinking water. Arsenic is observed to accumulate greater in paddy, green vegetables, and subterranean vegetables. Arsenic can also be found in fish, shellfish, pork, chicken, dairy products, and cereals; however, exposure from these foods is often considerably lower than exposure from contaminated groundwater [30]. The greatest risk to public health starts from contaminated groundwater. Arsenic in the form of inorganic ions is naturally found at higher levels in nearly 108 countries in the groundwater of many countries like Bangladesh, China, Mexico, Argentina, the United States of America, India, and Chile [31].

### 4.2. Mechanism of Toxicity

**Figure 4:**
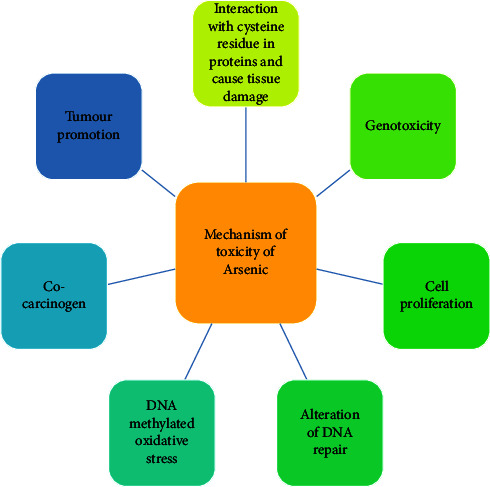
Mechanisms of toxicity of arsenic.

When arsenic enters the body through consumption, it is quickly absorbed into the bloodstream by the digestive tract. Arsenic in the blood binds to oxyhemoglobin and causes severe red blood cell death, limiting the blood's ability to carry oxygen to important organs and interfering with the kidney and liver's ability to function effectively [32]. Arsenic is a protoplastic toxin that specifically targets the sulphydryl group of cells, impairing cell enzyme function and cellular respiration [33].  Figure 4 shows the mechanism of toxicity of arsenic. Humans and organisms (fungi, bacteria, and algae) enzymatically methylate toxic inorganic arsenic compounds after chronic arsenic exposure to form less dangerous monomethylarsonic acid (MMA) and dimethylarsinic acid (DMA), which are expelled through urine. Monomethylarsonic acid (MMA III) is more toxic due to its high affinity for sulfhydryl groups. It cannot be excreted in the urine, and its accumulation is linked to arsenic-induced carcinogenesis [34].

### 4.3. Organ Toxicity and Associated Complications

Arsenic toxicity is known to cause anemia, gastrointestinal symptoms, skin lesions, neuropathy, hyperpigmentation, vascular lesions, liver damage, and renal damage [35]. Chronic arsenic poisoning has been shown to harm the cardiovascular system, neurological system, pulmonary system, endocrine system, and reproductive system. *T* cell and *B* cell functions are harmed by maternal arsenic exposure [36]. Vomiting, abdominal pain, and diarrhea are the first signs of acute arsenic poisoning. In severe cases, they are followed by numbness and tingling in the extremities, muscle cramps, and death [37].

Long-term exposure to arsenic can lead to malignancies of the bladder and lungs, in addition to skin cancer. Developmental impacts, diabetes, lung disease, and cardiovascular disease are some of the other negative health outcomes linked to long-term ingestion of inorganic arsenic. Myocardial infarction caused by arsenic, in particular, can be a major cause of death. Exposure to Arsenic has been related to “Blackfoot disease,” a severe illness affecting blood vessels and leading to gangrene [38]. Arsenic and phosphorus belong to the same periodic table group, and phosphate and arsenate chemicals have chemical similarities, allowing arsenic to substitute in key molecules or reactions such as DNA methylation disruption, DNA repair inhibition, oxidative stress disruption in ATP generation, and so on [39].

### 4.4. Treatment and Alternate System of Medicine

The first and foremost treatment of arsenic poisoning is dependent on the symptoms that have manifested; the patient must be removed from the source of exposure. In patients with significant burdens, chelating agents such as succimer and dimercaprol are essential. Hemodialysis should be considered for patients with kidney failure [40]. Plants and plant products have been used to treat ailments since the dawn of time. Plant-based medicine's main advantages appear to be perceived efficacy, low frequencies of serious side effects, and inexpensive cost. The current literature review found that medicinal plants like *Withania somnifera, Ipomea aquatica, Phyllanthus emblica, Triticum aestivum*, *Tephrosia purpurea, Camellia sinensis, Vitis vinifera, and Moringa oleifera* [41–43] and natural products like rutin, *β*-carotene, *α*-tocopherol, curcumin, resveratrol, and quercetin with arsenic toxicity alleviative effects simultaneously demonstrated a good intrinsic antioxidant effect by suppressing arsenic-induced oxidative stress through multimodal augmentation of endogenous defense mechanisms, resulting in arsenic toxicity amelioration [44, 45].

The natural compounds (phytochemicals) are well-known nutraceuticals and natural antioxidants. This demonstrates the need for antioxidant supplementation and backs up the suggestion for antioxidant therapy in humans. However, the biological advantages of these compounds must be confirmed in human subjects with chronic arsenic exposure. A potentized homeopathic medicine, Arsenicum album-30, was given to a group of arsenic patients, and the amount of arsenic in their urine and blood was measured regularly after that. The findings indicate that the medicine may be able to treat Arsenic poisoning in humans [46].

## 5. Mercury

### 5.1. Occupational Exposure and Sources

Mercury is a d-block element on the periodic table. It is the only common metal that is liquid at room temperature. It can exist in two oxidation states: I (mercurous) and II (mercuric). Inorganic, organic, and elemental forms of mercury show a slightly varied toxic profile, for instance, organomercuric compounds are considered most toxic owing to their ability to penetrate the BBB [47]. Gaseous mercury (used in electron tubes, argon lamps, etc.) is most difficult to control, owing to its high volatile nature. Dissolved elemental mercury, Hg (0), is widely observed in sediments and water which can get metabolized by microorganisms into organic mercury compounds such as methyl mercury which can then enter the food chain and accumulate [48].

Mercury emission can be from natural sources such as volcanic activity, weathering of rocks, oceans, soil, and biosphere accumulates, but the majority of the emission is due to anthropogenic activities such as combustion of coal, mercury-containing fuels [49], incineration of waste, mining, iron, and steel production. Global mercury emissions in 2000 were majorly from fossil fuel combustion which accounted for about 65%, gold production of around 11%, and nonferrous metal production and cement production with a mere 7% and 6% emission rate, respectively [50]. According to the WHO, a major cause of mercury exposure is due to dental amalgams and ingestion of contaminated seafood [51]. It is reported that carnivorous fish and animals have a higher chance of mercury toxicity than those of fishes and animals with herbivorous food habits [52]. Similar to what occurs in humans, higher animals (like mammals) can become poisoned by HM. The interaction of HM with invertebrates often results in genetic variation for tolerance and creates modifications in life-history traits. Invertebrates that have evolved to HM have shorter lifespans and make more effort during reproduction [53].

### 5.2. Mechanism of Toxicity

The two aspects that determine the toxicity of mercury are its solubility and its ability to form bonds with sulfhydryl groups. Primarily, the absorption rate of inorganic mercury into the body is around 2-38%, whereas organic mercury is almost completely absorbed into the bloodstream [54]. As aforementioned, it can enter the BBB and the CNS [55]. Also, the fat-friendly nature enables the toxin to enter the cell membrane conveniently. Being electrophilic, mercury tends to interact with nucleophile components such as sulfhydryl (–SH) or selenohydryl (–SeH) groups and forms stable complexes [56].

**Figure 5:**
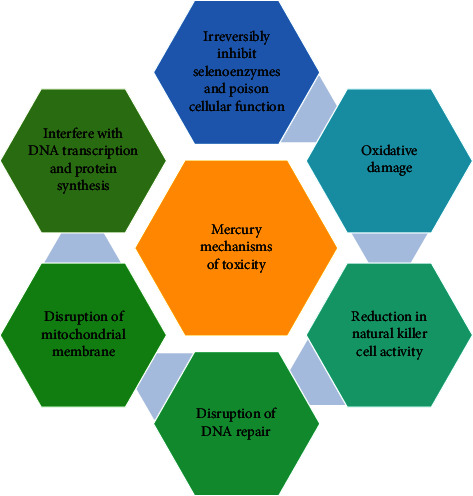
Mechanisms of toxicity of mercury.

The chemical interactions lead to the formation of ROS, thus inducing oxidative stress. ROS are known to cause cell death by damaging enzymes, lipids, and nucleic acids [57]. MeHg is also known to target and disrupt the mitochondrial electron transport chain and causes a reduction in the glutathione levels in the body by bonding with the thiol group in GSH and forming a GS-HgCH_3_ complex [56]. The free thiol group is particularly important for normal membrane functioning, and hence mercury blocks the physiological and metabolic functioning of the cell leading to oxidative stress and also damaging calcium homeostasis [58]. The blocking of the free thiol groups by mercury is one of the major causes of neurotoxicity and neuroinflammation. Apart from this, the tubulin protein of microtubules is also attacked by mercury compounds [59].  Figure 5 shows the mechanism of toxicity of mercury. It has been determined that mercury has deteriorated the effects of the antioxidant system and lipid peroxidation in addition to increasing the levels of superoxides. It has been reported that the signaling of the Nrf2-Keap1 molecules undergoes modifications. The DNA damage as a result of DNA breaks is one of the genetic alterations. Mitochondrial swelling, excessive H_2_O_2_ production, RER dilation, and alterations in the phosphorylation of JNK and GRP78 protein activation are a few noticeable ultrastructural changes [11].

### 5.3. Organ Toxicity and Associated Complications

The bonding of mercury with -SH groups is known to bring about secondary changes in the structure of DNA and RNA and structural changes in the ribosomal proteins [60, 61]. Mercury affects the activities of many neurotransmitters such as acetylcholine, serotonin, and norepinephrine [51]. It is shown to affect the immune system by inducing cytokine expression as well as IL-1 release [52] and also reducing the uptake of vitamin B12 and plasma ascorbic acid [52, 62]. Mercury is shown to induce immunosuppression, type-4 hypersensitivity, and autoimmunity by affecting cytokine production [63]. Prenatal exposure to organic mercury have been linked to mental retardation, deafness, blindness, and dysarthria in children [64].

Exposure to inorganic mercury on the other hand is shown to be associated with insomnia, weight loss, erythema, pruritus, excessive perspiration and hypersalivation, renal tubular dysfunction, and neuropsychiatry disorders [58]. There are reports of deposition of mercury in the kidney, liver, brain, and muscles. Mercury also has an affinity for *T* cell surface binding sites and sulfhydryl groups, which influences *T* cell function. Health effects of mercury include neurotoxicity, immunotoxicity, cardiovascular issues, carcinogenicity, and reproductive issues [65–67].

### 5.4. Treatment and Alternate System of Medicine

A few of the most used chelating agents for mercury toxicity are BAL, DMPS, DMSA, Penicillamine, Ca EDTA, etc. DMPS and DMSA are chelating agents that form complexes with various HMs including mercury. Much research suggests that administration with DMPS and DMSA resulted in increased urinary mercury output as compared to chelators such as BAL [68–70]. Iranmanesh et al. [71] have proved that the combined use of deferasirox and deferiprone is effective for treating mercury intoxication *in vivo*. DMSA and DMPS are administered orally and are known to effectively remove mercury through the kidney [71]. Additionally, DMSA is reported to be more efficient at the removal of methylmercury, including from the brain region [68, 72, 73]. Selenium plays a role in the metabolism of mercury in the body. Although the exact mechanisms remain unclear, selenium is shown to reduce the toxicity of mercury [74]. DMSA is considerably less toxic as compared to DMPS and Ca EDTA, which makes it the preferred chelating agent [75]. Despite being less toxic, these chelating agents possess numerous drawbacks. Adverse symptoms such as fever, muscle pain, and in severe cases difficulty in breathing, heart failure, and kidney damage are known to occur during the course of treatment and as a result, arises a need to find alternative natural sources of treatments [70, 75].

Owing to the side effects of chelation treatment, numerous other treatments have gained popularity in combination with chelation. In case of high toxicity, plasma exchange treatment can be used as an efficient alternative. It is most efficient for inorganic mercury poisoning. Induced sweating is found to be an effective treatment, while surprisingly hemodialysis was found to be ineffective [69]. The use of Ayurvedic medicine for the same is also gaining popularity [76]. The extracts of *Plathymenia reticulata, Connarus favosus* are shown to reduce acetylcholinesterase activity and methylmercury-induced lipid peroxidation, thus reducing mercury poisoning in zebrafish [77]. A recent study evaluated the protective effect of zinc (Zn) and N-acetylcysteine (NAC) on mercury toxicity in lactating rats [78]. Numerous natural antioxidants such as vitamin E, vitamin C, uric acid, and carotenoids are known to reduce the ROS released due to mercury toxicity [79–81].  Table 1 shows the health complications of HM toxicity.

## 6. Conclusion

The research efforts have increased the awareness related to HM-induced toxicity. Also, this review insists the important and effectiveness of alternative system of medicine. Nowadays, HM exposure has become a part of our day-to-day life, which cannot be avoided completely, but can be reduced significantly. This communication has thrown light on the adverse effects of these HMs. Even though modern medicines are effective in controlling HM-induced toxicities, which also cause severe side effects, they alter the normal metabolic process and sometimes extremely worsen the condition. However, alternative systems of medicine with little or no effects to treat the HM-induced toxicity will be the major focused area of research in the coming days. Plant products are the best sources to control metabolic disorders, including HM toxicity.

### 6.1. Recommendations

According to this communication, the recommendation is to go for alternative therapy for HM-induced toxicity than allopathic therapy because alternative system of medicine has few or no side effects.

## Figures and Tables

**Table 1:** Toxicity of lead, arsenic, and mercury.

Element	Route	Toxicity	Reference
Mercury	Inhalation	Deposited in the brain, thyroid, adrenals, skin, and pancreas and can impair the organs	[82, 83]
	Ingestion	Weakness, fatigue, anorexia, weight loss, and gastrointestinal disturbance	[83]
		Kidney targeting—abdominal pain, vomiting, and bloody diarrhea with potential necrosis of the gut mucosa	[84]

Arsenic	Ingestion of water or accidental ingestion of pesticides and insecticides	Vomiting, nausea, cyanosis, confusion, diarrhea, cardiac arrhythmia, and hallucinations	[85]
	Inhalation of arsenic gas	Shortness of breath, cough, bronchitis, lung cancer, chronic obstructive pulmonary disease, and bronchiectasis	
	Ingestion of water	Pigmentation of feet, hands, fingers, and keratosis	
		Central and peripheral vascular and cardiovascular disease, malignant diseases such as bladder, kidney, and liver cancer, diabetic millets, low blood count, and numbness	

Lead	Ingestion	Loss of neuron myelin sheath, reduction in the number of neurons, and it interferes with neurotransmission and decreases neuronal growth	[86]
		Adverse effects on certain organ systems like the central nervous system, the cardiovascular system, kidneys, and the immune system	[87]
		Affects osteoclasts and osteoblasts. Accelerates processes of bone formation and mineralization, which results in the formation of poor quality bones	[88]

## Data Availability

No data were used to support the study.
